# Allergen Immunotherapy (AIT) in children: a vulnerable population with its own rights and legislation – summary of EMA-initiated multi-stakeholder meeting on Allergen Immunotherapy (AIT) for children, held at Paul-Ehrlich-Institut, Langen, Germany, 16.1.2019 

**DOI:** 10.1186/s13601-020-00327-w

**Published:** 2020-06-29

**Authors:** V. Mahler, D. Mentzer, A. Bonertz, A. Muraro, P. Eigenmann, J. Bousquet, S. Halken, O. Pfaar, M. Jutel, U. Wahn, S. Vieths, S. Kaul

**Affiliations:** 1 Paul-Ehrlich-Institut, Paul-Ehrlich-Str. 51-59, 63225 Langen, Germany; 2 Food Allergy Referral Centre Veneto Region, Department of Women and Child Health, Padua General University Hospital, Padua, Italy; 3 Pediatric Allergy Unit, University Hospitals of Geneva, Geneva, Switzerland; 4 Charité, Universitätsmedizin Berlin, Humboldt-Universität zu Berlin, and Berlin Institute of Health, Comprehensive Allergy Center, Department of Dermatology and Allergy, Berlin, Germany; 5 University Hospital Montpellier, Montpellier, France; 6 MACVIA-France, Montpellier, France; 7 Hans Christian Andersen Children’s Hospital, Odense University Hospital, Odense, Denmark; 8 Department of Otorhinolaryngology, Head and Neck Surgery, Section of Rhinology and Allergy, University Hospital Marburg, Phillipps-Universität Marburg, Marburg, Germany; 9 Department of Clinical Immunology, Wrocław Medical University, Wrocław, Poland; 10 ALL-MED Medical Research Institute, Wrocław, Poland; 11 Pediatric Department, Charité, Berlin, Germany

**Keywords:** Allergen Immunotherapy (AIT), Children, Paediatric Investigation Plan (PIP), Therapy Allergen Ordinance (TAO), Paediatric Committee *(*PDCO), European Medicines Agency (EMA)

## Abstract

Concerning development of medicinal products, children belong to a so-called “special population” for which additional legislation applies: Regulation (EC) No 1901/2006 on medicinal products for paediatric use sets up a system of requirements, rewards and incentives to ensure that medicinal products are researched, developed and authorized to meet the therapeutic needs of children. Allergen Immunotherapy (AIT) is believed to contain a strong potential for immunomodulatory effects inducing sustained clinical efficacy after cessation of treatment (disease modifying effect) and thereby may prevent the progression of the atopic march towards asthma manifestation. However, to this day only few data on long-term effects in general exist and even fewer in children. These are predominantly data from open studies, which are strongly influenced in their validity by the known placebo effect of AIT. Furthermore, there are no studies allowing for the conclusion that efficacy in adults are mirrored by a similar efficacy in children and thus, up to now, it is not possible to extrapolate data from adults to children. The Paediatric Committee (PDCO)—European Medicines Agency’s (EMA) scientific committee responsible for activities on medicines for children—initiated a Multi-Stakeholder Meeting on AIT for Children held at the Paul-Ehrlich-Institut in Langen, Germany, to provide a platform for discussion and exchange of thoughts to this topic between allergy experts from academia, regulators and AIT-manufacturers. The consented meeting minutes, conclusions and participants are presented.

## Introduction

Concerning development of medicinal products, children belong to a so-called “special population” for which additional legislation applies: Regulation (EC) No 1901/2006 on medicinal products for paediatric use sets up a system of requirements, rewards and incentives to ensure that medicinal products are researched, developed and authorized to meet the therapeutic needs of children [1, 2]. Allergen Immunotherapy (AIT) is believed to contain a strong potential for immunomodulatory effects inducing sustained clinical efficacy after cessation of treatment (disease modifying effect) and thereby may prevent the progression of the atopic march towards asthma manifestation. However, to this day only few data on long-term effects in general exist and even fewer in children. These are predominantly data from open studies, which are strongly influenced in their validity by the known placebo effect of AIT. Furthermore, there are no studies allowing for the conclusion that efficacy in adults are mirrored by a similar efficacy in children and thus, up to now, it is not possible to extrapolate data from adults to children.

In 2008 the Therapy Allergen Ordinance (TAO) came into force in Germany to assure that all AIT products against frequent allergen sources, which were to this date on the market in Germany as Named Patient Products (NPP), are assessed for their benefit/risk-balance within a marketing authorization procedure [2]. For this purpose, for 123 AIT NPPs national marketing authorization applications were submitted in 2010. All these marketing applications for NPPs under the development plan of TAO had to be accompanied by a Paediatric Investigation Plan (PIP) approved by the Paediatric Committee (PDCO) of the European Medicines Agency (EMA) according to Article 7 of Regulation (EC) No 1901/2006. To simplify the assessment of 123 PIPs on short notice a Standard PIP for AIT products was developed.

According to Article 20 of Regulation (EC) No 1901/2006 PDCO [3] concluded that clinical trials in children as a vulnerable group may only be initiated after data on the efficacy and safety of the drug have been collected in adults and therefore according to Article 21 deferrals for the completion of clinical studies in children were granted in the PIPs. In addition, the PDCO determined that for children only the assumed disease modifying effect outweighs the risks associated with AIT. To prove long-term immunomodulatory effects a placebo-controlled blinded study design of 3-year-treatment with 2 years of follow-up is required and to be planned in the PIPs. However, the Standard PIP [Revision 4 (of February 2015)] [4] lays down that every manufacturer has to select ONE product (the so-called “selected product”) from his portfolio and to perform ONE long-term study in adults and in parallel—if necessary with a small time lag—ONE in children with this product to gain basic data for comparability and extrapolation purpose. After successful performance of these two studies with the ONE selected product the manufacturer can modify the PIPs for all other products of his portfolio and replace the long-term study with a short-term study.

PIP-compliance is a prerequisite for granting a marketing authorization not only for children, but also for the use in the adult population. To this point, however, none of the necessary AIT trials in children for the NPPs under the development plan of TAO have been initiated by the manufacturers, yet, raising increasing concerns in the future provision of licensed AIT products for children and adults.

PDCO therefore initiated [as follow-up meeting to a workshop on AIT for Children, held at EMA, 26th June 2018 (see Box 1)] a Multi-Stakeholder Meeting on Allergen Immunotherapy for Children held at the Paul-Ehrlich-Institut in Langen near Frankfurt, Germany, to provide a platform for discussion and exchange of thoughts to this topic between allergy experts from academia, regulators and AIT manufacturers [5]. The consented meeting minutes, conclusions and participants are presented.

**Box 1:** Short summary of outcomes from the first Workshop on AIT for Children, held at EMA, 26th June 2018 in London, UK

Aim of the first workshop: to exchange views with experts (from PEI, PDCO, EMA, EAACI nominated representatives and further external experts) on respiratory allergen immunotherapy in children, on the need for and feasibility of long-term studies
*General agreement among experts*
There is a relative need for more clinical studies to confirm sustained and long-term efficacy in children
At least 3 year treatment duration is required to elicit long-term effect (expert opinion based on clinical observation)
Three year placebo-controlled trials considered feasible, possible and acceptable (at least from investigator’s point of view—need to get parent’s view) for SLIT products; however considered unfeasible and unacceptable for SCIT products
Need to have same scoring systems in adults and in children among different manufacturers to allow future comparison of results not only between manufacturers but also between adults and children as basis for extrapolation—(agreed PIPs require the use of the combined score proposed by the EAACI Taskforce Group [6]) at least as secondary endpoint)
At present there is a lack of validated outcome measures for both adults and children. However, the combined symptom medication score (proposed by EAACI) has been used in well-powered studies in adults and children; this could be accepted as “validation” of this outcome
At present, there is no agreement on the most appropriate or optimal primary outcome measure to assess long-term efficacy of AIT for allergic rhinitis (AR) and allergic asthma (AA)
There is an urgent need to move to evidence generation and development of outcome measures which are not dependent on varying pollen exposure which is different from one season to the other
Manufacturers have been encouraged to explore the use of exposure chambers and compare results to field results, at least in adults—but not many manufacturers so far have followed this recommendation; consequently this comparison between field-exposure and exposure chambers is lacking at present
*Proposal to increase likelihood and feasibility of long-term studies in children*
For the PIP, it is recommended to separate SCIT and SLIT protocols
Proposal: First year double blind evaluation in field and in chamber, then open study and continue with chamber if results were comparable with those in double-blind period—possible to split comparison into 2 studies obviating need to do it in same study/same season?
Need to ask parents/patients about their expectations—what is important to achieve in term of resolution of symptoms, QoL, no need for medications (in order to improve adherence/compliance during the CTs)
A follow-up meeting with manufacturers, academia, EUnetHTA, regulators and parent/patient representatives should be considered to find solutions to the discussed proposals

## Multi-stakeholder Meeting^1^ on Allergen Immunotherapy (AIT) for Children

Date: 16 January 2019, Paul-Ehrlich-Institut, Langen, Germany.

This was a follow-up meeting on the workshop on AIT for Children (26th June 2018 at EMA, London,—see Box 1 for short summary).

The purpose of this follow-up meeting (with participation of several PDCO members) was to discuss with industry representatives, clinicians and investigators the proposals agreed at the June workshop with the aim to speed up initiation and conduct of paediatric clinical trials with those products.

### Background

The Therapy Allergen Ordinance in Germany (TAO, effective since November 2008) mandates a marketing authorisation (MA) for most prevalent therapy allergen products already on the market as named patient products (NPP), including sweet grasses, early flowering trees (birch, alder, hazel), house-dust mites and wasp or bee venom. 123 MA applications with an agreed PIP were received.

The requirements for the paediatric development plans as per the standard PIP for allergen products for specific immunotherapy, are:

- Per manufacturer: long-term studies in adults and paediatrics (normally 3 years treatment + 2 years follow-up) double-blind, placebo-controlled for ONE product (selected product).
- Selected product for these studies to be chosen by the manufacturer.
- For all other products from the manufacturer (prior to successful conclusion of long-term studies with selected product) long-term studies in paediatrics are required.
- After successful long-term studies with the selected product, manufacturer can choose duration of clinical trials for other products (via PIP modification).
- Studies in children are supposed to start as soon as short-term efficacy and safety has been proven in adults.

As of January 2019, 65 out of the initial 123 applications by 7 manufacturers are still in development, partly due to considerable challenges with the clinical development in adults:

- Dose-finding studies (DFS) are still lacking for a considerable number of products.
- Several DFS were inconclusive.
- According to the results of DFS, for some products a higher dose than currently marketed as NPP suggests a better benefit-risk-balance. Yet, for most of these products convincing phase III study data are still lacking.
- Until now no currently marketed dose was confirmed in a valid DFS.
- To date, only two products have received a marketing authorisation for a higher dose than originally marketed—however, until now, no study in children was performed and thus the products are only indicated in adults.

As a consequence the clinical development in adults is hugely delayed. As according to current legislation and the standard PIP paediatric studies should only start after confirmation of short-term efficacy in adults, the start of paediatric trials is also seriously delayed.

As of today, one manufacturer fulfilled the requirement regarding long-term studies with the selected product in adults and children (selected product not being subject to TAO); for 6 selected products long-term studies in adults and children are still needed. Until now no studies in children were/are performed with products being subject to TAO.

Although the main reason for the delay in initiating paediatric studies is due to the major challenges with dose finding for adults, allergen manufacturers repeatedly point out that the requirements for long-term studies (both in adults and particularly in children) are a too high hurdle and the requested studies are not feasible for several reasons, including unwillingness or ethical concerns of patients/parents/investigators to enrol into a 3-year placebo-controlled study with 2 years blinded follow-up, and the high administrative and financial burden of conducting large long-term studies.

In addition, it was mentioned that due to the availability of alternative products on the market, patients/parents are not willing to participate in a trial in which the child has the chance to receive placebo. Moreover, the risk of long-term study failure is too high as there are currently no validated standardized endpoints for children and there is insufficient knowledge regarding patient eligibility criteria. Initiating a long-term trial before these gaps have been resolved is therefore considered to be neither ethical nor meaningful.

Following this comment disappointment was expressed that after so many years of use of AIT in the currently marketed but not authorised way these well-known gaps in knowledge have been not yet been adequately addressed.

The discussion focused on three main issues:

### 1. Paediatric study design

Among clinicians and investigators there was consensus that long-term studies are considered feasible in children with sublingual immunotherapy products (SLIT) but not with subcutaneous immunotherapy (SCIT) products; the requirement for placebo injection for 3 years within SCIT trials is seen as a too high burden;

The following suggestions by clinicians and/or industry representatives were made:

- To keep children only for 1 year in the placebo arm; only possible once validated innovative endpoints independent of allergen exposure are available.
- To investigate products from different companies combined (e.g. 2–3 birch pollen products) using one joint placebo arm (industry representatives did not comment this proposal by investigators).
- Start with 1-year trial in adults and in children to generate evidence that the product is safe and efficacious, and only then do a 5-year, potentially combined (several different active arms with only one joint placebo–arm) trial.
  Difficulty: once the product is on the market, it will no longer be feasible to conduct a long-term study.
- Combined adult/adolescent and children (< 12 years of age) studies; this has the disadvantage that in the latter age group not the children themselves but the parents do the scoring. This raises the question if results scored by the parents for the younger children are comparable with those generated by adolescent or adult patients themselves.
- Need for input from patient/parent what they consider relevant in terms of disease control (symptom control, decrease in rescue medication use, …).
- The use of real world data instead of prospective placebo-controlled randomised studies; Problem: data collection would have to fulfil regulatory quality standard; at present, this is not the case.
- To consider active controlled studies;

However, non-inferiority studies would require even larger study population, and up to now no authorised comparator is available for many allergen groups.

- It was proposed to perform safety studies only in children, as for all products on the market the dose for adults and children is the same, and there is currently no indication that the efficacy is lower in children than in adults. Based on the available studies experts concluded that in general even a higher efficacy is generally observed in children. However, the hypothesis, that the efficacy is equal or higher in children as in adults needs yet to be proven to reduce the current uncertainty of knowledge.

### 2. Endpoints, alternatives to medication and symptom scores

- Need for harmonised endpoints to allow comparison of various studies of different manufacturers, and between adults and children.
- The combined symptom medication score developed by the European Academy of Allergology and Immunology (EAACI) has been used in well-powered studies in adults; however, the score is not validated (also not for adults) and usefulness for children has to be shown in clinical trials.
- Need for outcome measures which are not dependent on varying pollen exposure being different from one season to the other, e.g. exposure chambers for primary endpoint analysis.
- An EAACI task force is working on a proposal for a hybrid study design to compare exposure chamber with field study results (recommendation for technical specifications and validation have been previously published).
- Precompetitive collaboration among manufactures is suggested for validation of new, innovative endpoints, such as the use of allergen challenge chambers.
- Manufacturers are encouraged
  - to explore the use of exposure chambers and compare results to field results, at least in adults.
  - to seek regulatory qualification advice for new outcome measures and use of exposure chambers.
  - to collaborate among themselves to explore new biomarkers (as endpoint or for prediction/therapy decision), study designs, study networks and others.
- Need for harmonised and agreed appropriate endpoints to assess long-term efficacy of AIT on prevention of/impact on allergic asthma.

### 3. Timing of initiation of paediatric studies

- Clarification of current standard PIP requirement:
  - Manufacturers have the right to select ONE allergen product out of their portfolio for the long-term studies in adults and children and initiate long-term study in children as soon as dose–response and first efficacy (e.g. after one year) and safety data in adults are available. Manufacturers are not obliged to conduct long-term studies for both SCIT and SLIT products;
  - Manufacturer may submit data to regulatory agencies in between for the purpose of marketing authorisation provided PIP compliance is fulfilled, e.g. once 3-year treatment period is completed for sustained efficacy while the trial is continued with blinded treatment-free period of 2 years. Regulatory assessment of submitted data will be performed in parallel to the proceeding trial; hence no need to wait until full 5 year study period is completed.
  - During the 2 year treatment-free follow-up period study participants and investigators to remain blinded for the treatment allocation during the 3-year treatment period.
  - There is no need for DFS in children.
- Manufacturers were informed that EAACI is establishing a network of independent sites with the capacity to conduct large (paediatric) clinical studies.

Finally the discussion also touched upon extrapolation:

- Clinicians and investigators stressed the need for evidence of safety and efficacy;
- Such evidence should mainly be generated in adults.
- There was agreement among regulators and investigators that at this point in time extrapolation is not yet possible, as evidence in the source population, i.e. adults, is still missing.
- Extrapolation of efficacy data from adults and perform only safety studies in paediatrics is only possible after scientific proof of concept that extrapolation from adults to paediatrics is feasible. This proof may be performed in SLIT products.
- Once long-term efficacy of SLIT products has been convincingly demonstrated in adults and children, generating evidence from long-term efficacy of SCIT products only in adults may be sufficient to use for extrapolating long-term efficacy in the paediatric population.

### 4. General information about pending PIP revisions of TAO products

The timelines of the originally approved PIPs of the TAO products are running out in most cases. Thus, a PIP revision is necessary for nearly all companies to be PIP compliant.

- PIP compliance is mandatory to start MAA assessment.
- Most companies dealing with TAO products need a PIP-revision agreed by PDCO to be PIP compliant (at least the timelines of the paediatric trials have to be modified in the approved PIPs).
- Changes in the PIP opinion (re timelines) will be accepted by PDCO once a long-term efficacy (LTE) trial in the paediatric population (aged 5–12 years) has been initiated; the earliest time point for this modification of a PIP opinion is first paediatric patient passed first visit (definition by Irmgard Eichler, EMA).

This means: no TAO product will pass future compliance check before at least the LTE of the selected allergen product has been started in children. (As stated above: Studies in children are supposed to start as soon as short-term efficacy and safety has been proven in adults).

- Paediatric trials (STE or LTE) can be started at any time, provided the trial is compliant with the key binding elements (KBE) in the PIP opinion. (as stated above).
- Provided PIP compliance is confirmed, each TAO product can go for MAA for its individual indication.

## Conclusions

PDCO to discuss the various suggestions and to consider potential changes of the standard PIP.

EAACI and manufacturers invited to propose alternative approaches for development of SCIT products.

## Next steps

- Based on feedback from investigators, no change considered necessary for paediatric development of SLIT products.
- PDCO will await proposals from EAACI and manufacturers how to investigate long-term efficacy and safety for SCIT products.
- PDCO supports multi-company studies on the same allergen class with one common placebo arm.
- PDCO encourages precompetitive collaboration to increase knowledge base.

## Data Availability

Not applicable.

## Abbreviations

AIT: Allergen Immunotherapy
CT: Clinical trial
DFS: Dose finding study
EAACI: European Academy of Allergy and Clinical Immunology
EC: European Commission
EMA: European Medicines Agency
EUnetHTA: European Network for Health Technology Assessment
KBE: Key binding elements
LTE: Long-term efficacy
MA: Marketing authorisation
MAA: Marketing authorisation application
NPP: Named patient product
PDCO: Paediatric Committee (of EMA)
PIP: Paediatric Investigation Plan
QoL: Quality of Life
SCIT: Subcuteaneous immunotherapy
SLIT: Sublingual immunotherapy
STE: Short-term efficacy
TAO: Therapy Allergen Ordinance
TF: Task force

## Footnotes

1 Participants:

- Nominated stakeholders: Birgit Ahrens (PEI), Sophia Aldous (ROXALL Medizin), Silke Arzberger (Lofarma Germany GmbH), Nicole Armbrüster (BPI), Andreas Bonertz (PEI), Albrecht Bufe (Ruhr-Universität Bochum), Caroline Dorrepaal (HAL Allergy), Irmgard Eichler (EMA), Gabriele-Cornelia Fox (Allergopharma), Marek Jutel (EAACI), Efstrathios Karagiannis (Stallergens GmbH), Susanne Kaul (PEI), Stephan Kerkojus (Lofarma Germany GmbH), Denise Lee (ATL), Vera Mahler (PEI), Dirk Mentzer (PEI, PDCO), Marek Migdal (PDCO), Antonella Muraro (EAACI), Dirk-Jan Opstelten (HAL Allergy), Oliver Pfaar (EAACI), Johanna Rost (PEI), Sabine Scherer (PDCO), Lindsay Schüler (Bencard Allergie GmbH), Hugo Tavares (PDCO), Eva-Cornelia Ticinelli (LETI Pharma GmbH), Jenny Uhlig (ROXALL Medizin), Ulli Umpfenbach (Charité Universitätsmedizin Berlin), Stefan Vieths (PEI).
- Excused nominated stakeholders: Susanne Lau (Charité Universiätsmedizin Berlin), Andy Möckel (LETI Pharma GmbH), Francesca Rocchi (PDCO).
- Further participants: Jean Bousquet (EAACI TF on Clinical Trials in Children), Philippe Eigenmann (EAACI TF on Clinical Trials in Children), Susanne Halken S (EAACI TF on Clinical Trials in Children), Ulrich Wahn (EAACI TF on Clinical Trials in Children).
